# Prospective estimation of water equivalent diameter and SSDE using 3D landmark CT scans

**DOI:** 10.1002/acm2.70742

**Published:** 2026-08-24

**Authors:** Andrew M. Hernandez, Sarah Yoon, Michael T. Corwin, Eric S. Diaz, Ahmadreza Ghasemiesfe, Sarah E. McKenney, John M. Boone

**Affiliations:** ^1^ Department of Radiology University of California Davis Health Sacramento California USA; ^2^ Department of Biomedical Engineering University of California Davis Davis California USA

**Keywords:** 3D landmark, radiation dose, size‐specific dose estimate, water equivalent diameter

## Abstract

**Background:**

3D Landmark (3DLM) scans have recently been introduced in some new CT scanners for automatic scan planning instead of paired 2D localizers, with potential additional utility.

**Purpose:**

The purpose of this work was to measure the accuracy and precision of 3DLM scans for determining water equivalent diameter ($D_{w}$) and size‐specific dose estimate (SSDE) in patient CT exams.

**Methods:**

A cohort of 223 diagnostic CT series comprised of 82 Head [52 non‐contrast (NC); 30 contrast‐enhanced (CE)], 66 Chest (36 NC; 30 CE), and 75 Abdomen‐pelvis (45 NC; 30 CE) series, acquired on a CT scanner equipped with 3DLM capabilities, were retrospectively collected. All series were acquired for patient standard‐of‐care, and 3DLM series were generated according to the manufacturer's default protocol. $D_{w}$ values were measured on the central axial image (along z) of each CT series and 3DLM series, using established methods, and were independently used to derive SSDE. Accuracy and precision of 3DLM series relative to reference NC and CE series were assessed for both $D_{w}$ and SSDE using Bland‐Altman analysis and Wilcoxon signed‐rank test across the three body regions. Root mean square error (*RMSE*) of $D_{w}$, computed across series within each protocol, and absolute relative error (*ARE*) of SSDE, computed per series, were compared against AAPM‐established clinical acceptability thresholds (10% per TG‐220; 20% per TG‐204/293). Proportional bias with patient size ($D_{w}$) was evaluated using ordinary least squares regression. Per‐series differences in $\;D_{w}$ and SSDE bias between NC and CE series were assessed within each body region using the Mann‐Whitney U test. SSDE determination performance was compared between the new [SSDE(3DLM, $D_{w})$] and conventional [SSDE(AP/LAT, $D_{w}$)] methods using the Wilcoxon signed‐rank test to assess differences in bias (accuracy) and Levene's test to assess difference in error variability (precision).

**Results:**

The mean [range] patient age was 45.9 years old [0.1–91.5]. The range of $D_{w}$ for the reference NC/CE series was 12.6 to 20.9 cm for Head, 9.8 to 35.6 cm for Chest, and 12.7 to 41.6 cm for the Abdomen‐pelvis. The $D_{w}$ *RMSE* per protocol and SSDE *ARE* per series were < 3% and < 7.1%, respectively, substantially below AAPM‐established clinical acceptability thresholds. A statistically significant bias in $D_{w}$ and SSDE was identified for the Head and Chest (*p *< 0.001 for all comparisons) but the median biases were small ($D_{w}$ range: −0.6% to −2.1%; SSDE range: (0.4% to 1.9%). Unidirectional $D_{w}$ underestimation and SSDE overestimation was observed only for the Head protocols. $D_{w}$ estimation bias showed negligible dependence on patient size (R^2 ^< 0.185) across all protocols. $D_{w}$ and SSDE bias differed significantly between the NC and CE series in the Head (*p*_Mann‐Whitney_ < 0.001) and Chest (*p*_Mann‐Whitney_ < 0.05). SSDE(3DLM, $D_{w}$) provided significantly lower median bias compared to SSDE(AP/LAT, $D_{w}$) for the Head and Chest (*p* < 0.001), as well as lower per‐case variability in error across all protocols (*p_Levene_* < 0.001).

**Conclusion:**

3DLM‐based measurements of $D_{w}$ and SSDE achieved clinically robust accuracy and precision relative to the reference standard, with SSDE(3DLM, $D_{w}$) also outperforming the conventional AP/LAT‐derived method.

## 1 INTRODUCTION

Some newer model CT scanners utilize a combination of an ultra‐low dose 3D landmark (3DLM) scan and AI‐based Automatic Landmark Detection that eliminate manual scan planning by automatically identifying the anatomical limits of the scan region. This automatic approach has been shown to improve clinical workflow by reducing the need for manually defining the scan region^1^ and to reduce acquisition range in chest, abdomen, and chest‐abdomen‐pelvis CT scans.^2^ A Monte Carlo study also found that 3DLM scans result in lower effective dose in chest and abdomen CT scans compared to traditional 2D localizers.^3^

The size‐specific dose estimate (SSDE) is now a well‐established metric for estimating patient dose in CT that accounts for patient size. Consensus Task Group reports have been published by The American Association of Physicists in Medicine (AAPM) that provide conversion factors for body and head exams, respectively. These factors can be used to compute the SSDE from scanner‐reported CTDI_vol_ and patient size. AAPM TG Report 220 introduced the concept of water equivalent diameter ($D_{w}$) for a robust patient size metric, that directly accounts for patient attenuation. The International Electrotechnical Commission has incorporated the SSDE and $D_{w}$ into its dosimetry standard, IEC 62985:2019, requiring new scanners to report these metrics.

Retrospective CT image‐based estimations of $D_{w}$ and hence SSDE are useful for patient dose monitoring, for establishing diagnostic reference levels, and for local dose comparisons against regional and national benchmarks. Ideally, these metrics can be prospectively estimated prior to the CT acquisition for automated, task‐based optimization of the imaging protocol and resulting dose to the patient. Methods have been introduced to measure $D_{w}$ from the CT localizer radiographs but some implementations can be inaccurate if the patient is not well positioned, and accurate estimations require two orthogonal localizers.^4^ Estimating accurate $D_{w}$ values from 2D localizers also requires a complex calibration process that correlates pixel values in the localizer with x‐ray attenuation,^5^, ^6^ and the stability of this calibration over time remains poorly understood.^4^ 3DLM scans are three to four times faster than acquisition two orthogonal 2D localizers^1^ and CT images reconstructed from the 3DLM acquisitions are already calibrated in terms of attenuation as part of routine system calibration. These capabilities motivated the present study, which aimed to evaluate whether 3DLM scans can be used beyond their intended purpose to provide accurate, prospective estimation of patient $D_{w}$ and SSDE in clinical CT examinations.

## 2 METHODS

### 2.1 Imaging platform and acquisition parameters

All evaluated images were acquired on the Aquilion One Insight Edition (Canon Medical Systems Corporation, Otawara, Japan) CT scanner at our institution. Default acquisition and reconstruction parameters for the 3DLM scans were utilized in this work as detailed in Table 1 unless otherwise specified. All diagnostic non‐contrast (NC) and contrast‐enhanced (CE) scans included in this study were generated according to our institution's clinical protocol and acquired as a part of the patient's standard‐of‐care. This retrospective study of standard‐of‐care images received an exemption determination from the Institutional Review Board.

**TABLE 1:** Default acquisition and reconstruction parameters used for 3D Landmark (3DLM) scans.

**Tube voltage**	120 kV
**Filtration material**	Ag
**Fixed tube current**	20–50 mA
**Rotation time**	0.5 s
**Collimation width**	80 × 0.5 mm
**Pitch**	1.388
**Recon algorithm; denoising level**	AIDR 3D; L4
**Recon kernel**	Body
**Display FOV**	500 mm
**Voxel size**	∼1 mm isotropic

To evaluate potential beam quality differences between the 3DLM Ag filtration and the Cu filtration used for the diagnostic CT acquisitions, TASMICS^7^ was used to model the 120 kV/Cu and 120 kV/Ag x‐ray spectra using previously‐reported Ag and Cu filter thicknesses at 120 kV.^3^ The resulting half‐value layer and effective energy for each modeled spectrum were then computed. Full modeling parameters and the resulting x‐ray spectra are provided in the Supplemental Material.

### 2.2 Patient population

The Institutional Review Board at our institution approved this retrospective study with a waiver for informed consent. Patient exams were retrospectively identified from November 2024 to September 2025 through periodic sampling of standard‐of‐care CT exams. Acquisitions were categorized into six protocols defined by body region (Head, Chest, Abdomen‐pelvis) and contrast administration (non‐contrast [NC] and contrast‐enhanced [CE]). Head CT acquisitions with patient arms positioned above the head or any Chest or Abdomen‐pelvis acquisitions with arms positioned down were also excluded from the study.

Two CE Head CT protocols were evaluated: CT Angiography (CTA) Head and CT Head with contrast. CTA Head was acquired as a single arterial‐phase acquisition. CT Head with contrast was acquired as a single delayed‐phase acquisition. Three CE Chest CT protocols were evaluated: CTA Chest, CTA Chest for Pulmonary Embolism, and routine CT Chest with contrast. CTA Chest and CTA Chest PE were each acquired as a single arterial‐phase acquisition, and the routine Chest protocol was acquired at a late arterial phase. Three CE Abdomen‐pelvis CT protocols were evaluated: CT Abdomen‐pelvis for Hemorrhage, CTA Abdomen‐pelvis, and routine CT Abdomen‐pelvis with contrast. CT Abdomen‐pelvis for Hemorrhage is a multiphase protocol. The arterial phase was analyzed and delayed, and venous phases were excluded. CTA Abdomen‐pelvis was acquired as a single arterial‐phase acquisition. The routine CT Abdomen‐pelvis with contrast protocol was acquired as a single portal venous‐phase acquisition.

The periodic sampling required manually sending the default 3DLM series from the scanner to the PACS used in this study as automated 3DLM series transferring was not an available setting. Where necessary, a larger field‐of‐view was retrospectively reconstructed for the selected NC and/or CE series to ensure complete anatomical coverage in the central axial plane for accurate $D_{w}$ measurements as described in the following sections. For each acquisition, the scanner‐reported anterior‐posterior (AP) and lateral (LAT) body size estimations were recorded from each dose summary sheet and used to compute the effective diameter: $D_{eff}=\sqrt{AP\times LAT}$.

### 2.3 Water equivalent diameter

For each NC and CE acquisition, $D_{w}$ was measured in the central axial plane of the series with the largest slice thickness and smoothest reconstruction kernel. All measurements were performed using ROI annotation tools available on the PACS following established AAPM guidelines.^5^ The slice thickness of the corresponding 3DLM series was adjusted to match the diagnostic series using the slice averaging functionality available on the PACS. The same physical slice location corresponding to the central axial slice of the CE/NC series was chosen for $D_{w}$ measurements in the 3DLM series. In a small number of cases where patient motion between acquisitions resulted in considerable anatomical misalignment, the reference slice for the 3DLM‐based $D_{w}$ estimation was adjusted to maintain consistent anatomical positioning with the corresponding NC and/or CE series. The study author responsible for placing ROIs for all measurements, and in some cases adjusting the references slices, was blinded time to downstream $D_{w}$ calculations at the time of measurement.

### 2.4 SSDE

All $D_{w}$ measurements were then used to calculate the size SSDE using the α and β fit parameters which describe the CTDI_vol_‐to‐SSDE conversions factors “f” reported by AAPM Task Group 204 for Body exams ($f^{B}$)^8^ and TG 293 for Head exams $(f^{H}$)^9^ as a function of $D_{w}.$ This resulted in SSDE estimations from 3DLM‐based $D_{w}$ measurements “$SSDE(3DLM,D_{w}),$” and SSDE estimations from NC‐ and/or CE‐based $D_{w}$ measurements “$SSDE{\left(NC/CE,D_{w}\right)}^{\prime \prime }.$”

For comparison, SSDE was also estimated using conventional scanner‐reported anterior‐posterior (AP) and lateral (LAT) size metrics “SSDE(AP/LAT, $D_{w}$)”. This method requires calculating $D_{eff}$ from the AP/LAT values and then, if applicable, converting to $D_{w}$. The $f^{B}$ conversion factors were estimated from the TG‐204 reported fit parameters using $D_{eff}$ directly as the authors of TG‐220 note that no corrections are required when substituting $D_{w}$ for $D_{eff}$.^5^ The $f^{H}$ conversion factors in TG‐293 are only reported as a function of $D_{w}$ values. Therefore, to compute $D_{eff}$‐derived $D_{w}$ values for the head, the following linear relationship reported by Fahmi *et al.* ^10^ for pediatric head CT was used: $D_{w}=$1.0962$\times D_{eff}-0.7768$.

### 2.5 Statistical analysis

$D_{w}$ measurements and derived SSDE values from 3DLM and reference diagnostic CT series were compared at the series level, with relative error, calculated as (3DLM—reference)/reference and expressed as a percentage, reported for each comparison. Compliance with AAPM TG‐220 recommendations^5^ was assessed by computing the root mean square error (RMSE) across all series within a given protocol (e.g., NC Head), with a value less than 10% considered clinically acceptable for $\;D_{w}$ measurements within that protocol. For SSDE, clinical acceptability was defined per series using the absolute relative error (ARE), calculated as the absolute value of the relative error described above, with values less than 20% considered acceptable, consistent with reported in AAPM TG‐204^8^ and TG‐293.^9^ Statistical analysis consisted of two main components: (1) Bland‐Altman analysis to assess systematic bias, as a measure of accuracy, and limits of agreement, reflecting error variability (i.e., precision); and (2) evaluation of proportional bias with respect to patient size.

Differences were first tested for normality using the Shapiro‐Wilk test. Non‐normal $D_{w}$ and SSDE error distributions were identified in select protocols. Therefore, the Wilcoxon signed‐rank test was used across all protocol and metric comparisons, and both the mean and median biases are reported herein with both the Bland‐Altman analysis (described below) and Wilcoxon significance testing. Effect size was quantified using rank‐biserial correlation *r*.^11^

Bland‐Altman analysis was used to assess accuracy and precision in $\;D_{w}$ and SSDE measurements between the prospectively acquired 3DLM series and the reference diagnostic CT datasets, using the relative error plotted against the reference (diagnostic CT) value.^12^ Mean bias and 95% limits of agreement (LoA) were calculated as mean relative error ±1.96×SD. The 95% confidence intervals were computed for the mean bias and LoA using the t‐distribution with n‐1 degrees of freedom, with standard errors of $\sqrt{SD^{2}/n\;}$ for the bias and $\sqrt{3\times SD^{2}/n\;}$ for the LoA following Bland and Altman.^12^, ^13^ In addition, differences in per‐series $\;D_{w}$ and SSDE error between NC and CE series were assessed within each body region using the Mann‐Whitney U test.

To assess proportional bias with patient size, a linear regression model describing the error in $\;D_{w}$ estimations as a function of the reference (diagnostic CT)$\;D_{w}$ values was fit for each body region using ordinary least squares. A statistically significant slope and high coefficient of determination (R^2^) would indicate that patient size ($D_{w}$) is a statistically significant and strong predictor of estimation error.

SSDE determination performance was then compared between the new [$SSDE(3DLM,D_{w})]$ and conventional [SSDE(AP/LAT, $D_{w}$)] methods with all NC and CE series pooled within each body region. To assess whether accuracy differed between the two methods, per‐series SSDE error (bias) was compared using the Wilcoxon signed‐rank test. To assess whether precision differed between the two methods, per‐series SSDE error variability, quantified as standard deviation (SD), was compared using Levene's test for equality of variance.

For all statistical tests, a *p*‐value less than 0.05 was considered statistically significant. P values were reported simply as “*p”* for all Wilcoxon tests, as “*p*_Mann‐Whitney_” for Mann‐Whitney U tests, and as “*p*_Levene_” for Levene's test.

## 3 RESULTS

### 3.1 Patient population

**FIGURE 1:**
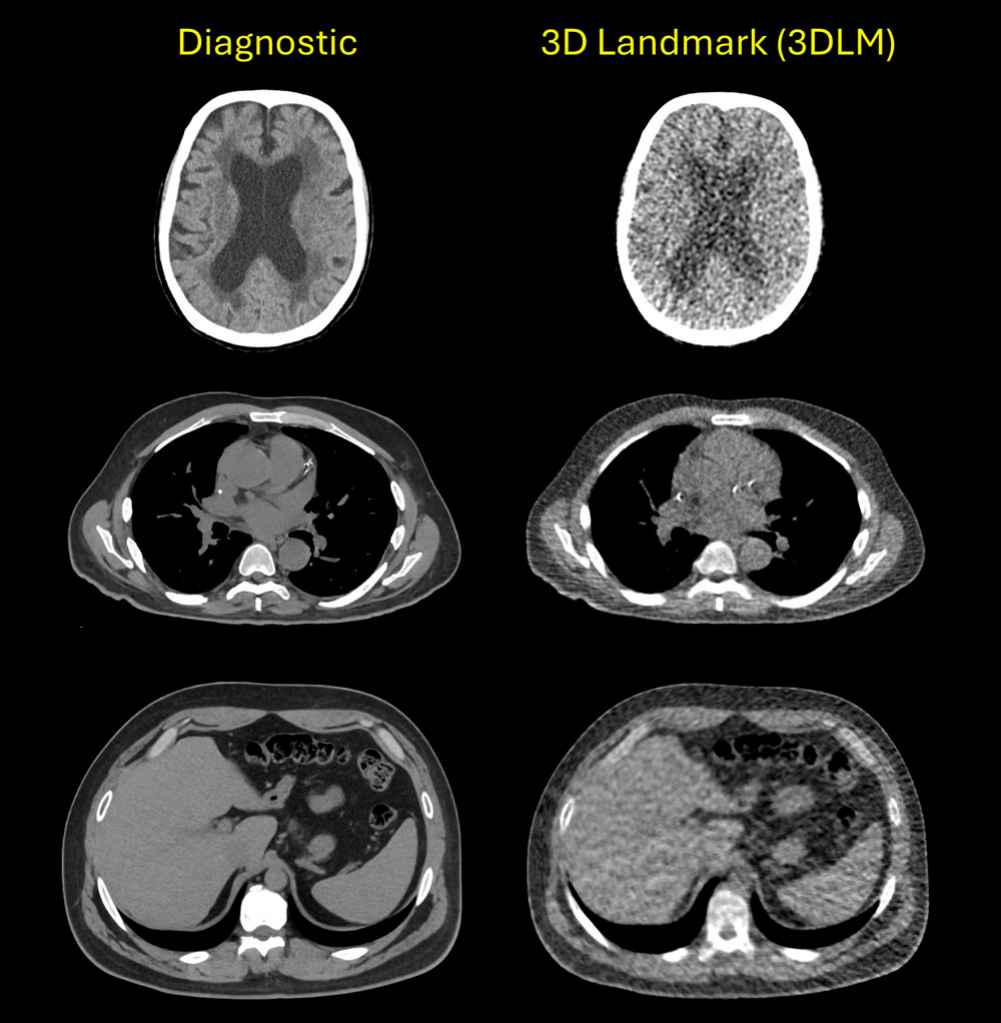
Example central axial slices through diagnostic non‐contrast (NC) CT and 3D landmark (3DLM) scans for Head, Chest, and Abdomen‐pelvis body regions. All images are shown with the same soft tissue window/level setting (W/L = 400/40).

A total of 191 unique exams with at least one NC or CE acquisition were included in this retrospective study totaling 223 CT series (82 Head, 66 Chest, and 75 Abdomen‐pelvis). Details of the patient population as well as $D_{w}$ values and SSDEs from the diagnostic CT scans are included in Table 2. Figure 1 depicts examples of central axial slices through NCCT and 3DLM series for all three body regions. In nine of the 223 series (4.0%), the reference slice location required manual adjustment from the automatically selected 3DLM slice.

**TABLE 2:** Summary of patient population, size metrics, and size specific dose estimates included in this study.

# of Head series [NC; CE]	82 [52; 30]
# of Chest series [NC; CE]	66 [36; 30]
# of Abd‐pel series [NC; CE]	75 [45; 30]
# of unique patients [pediatric < 19 y.o.]	180 [38]
Patient age: mean (y.o) [range]	45.9 [0.1–91.5]
$D_{w}$: mean (cm) [range]	NC/CE Head: 18.2 [12.6–20.9] NC/CE Chest: 25.4 [9.8–35.6] NC/CE Abd‐pel: 28.6 [12.7–41.6]
SSDE: mean (mGy) [IQR]	NC/CE Head: 42.2 [16.7] NC/CE Chest: 10.9 [6.8] NC/CE Abd‐pel: 5.8 [6.3]

Abbreviations: CE, contrast‐enhanced; $D_{w},$ water‐equivalent diameter; IQR, interquartile range; NC, non‐contrast; SSDE, size specific dose estimate; y.o., years old.

### 3.2 Water equivalent diameter

Table 3 summarizes the statistical analysis results for $D_{w}$ estimations. Each of the six protocols achieved RMSE values below the 10% TG‐220 compliance threshold (range: 1.3%–2.9%), confirming clinically acceptable $D_{w}$ estimation using the 3DLM series for all body regions, with and without contrast. A statistically significant bias (*p *< 0.001) was identified in 2 of the 3 body regions (Head and Chest), while no significant bias was observed for the Abdomen‐pelvis body region. The effect size was also uniformly large for the Head and Chest body regions with rank‐biserial *r* ranging from 0.63 to 0.87, and negligible for the Abdomen‐pelvis body region (*r* range: 0.02–0.04).

**TABLE 3:** Accuracy (bias) and precision (limits of agreement) of water equivalent diameter ($D_{w}$) measurements from 3D landmark relative to the reference diagnostic CT series, summarized by protocol.

Protocol	Mean Bias [95% CI] (%)	Median Bias (%)	*p*‐value^*^	Effect Size	RMSE (%)	Lower LoA [95% CI] (%)	Upper LoA [95% CI] (%)
**NC Head**	−1.2 [−1.4, −1.0]	−1.1	<0.001	0.87	1.3	−2.4 [−2.7, −2.1]	0.0 [−0.3, 0.3]
**CE Head**	−2.0 [−2.4, −1.7]	−2.1	<0.001	0.87	2.3	−4.0 [−4.6, −3.4]	−0.1 [−0.7, 0.5]
**NC Chest**	−0.8 [−1.2, −0.4]	−0.6	<0.001	0.59	1.5	−3.2 [−3.9, −2.5]	1.6 [0.8, 2.3]
**CE Chest**	−1.8 [−2.7, −0.9]	−2.1	<0.001	0.63	2.9	−6.4 [−8.0, −4.9]	2.9 [1.3, 4.4]
**NC Abd‐pel**	−0.1 [−0.6, 0.4]	−0.0	0.911	0.02	1.7	−3.4 [−4.3, −2.5]	3.2 [2.3, 4.1]
**CE Abd‐pel**	−0.2 [−0.7, 0.4]	0.2	0.839	0.04	1.4	−3.0 [−3.9, −2.0]	2.7 [1.7, 3.6]

Abbreviations: CE, contrast‐enhanced; CI, confidence interval; LoA, limits of agreement; NC, non‐contrast; RMSE, root mean square error.

* Test whether median $D_{w}$ bias differs significantly from zero using the Wilcoxon signed‐rank test.

**FIGURE 2:**
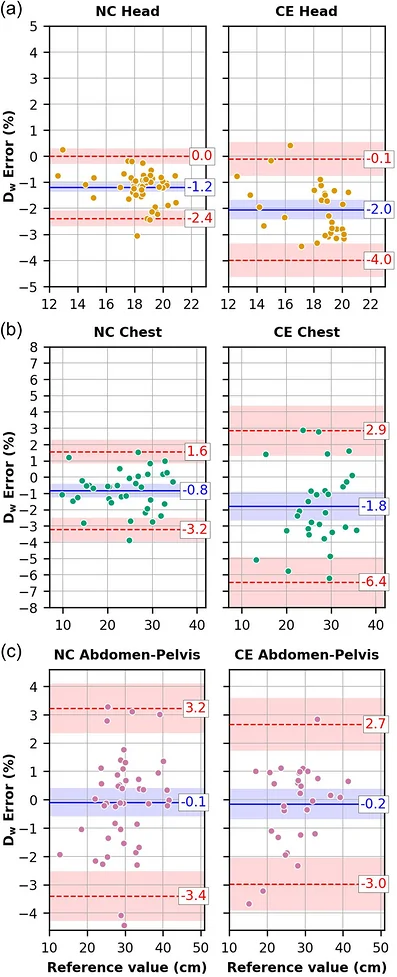
Bland‐Altman plots depicting water‐equivalent diameter ($D_{w})$ relative error, expressed as a percentages, for the 3D landmark (3DLM) method compared to reference diagnostic CT measurements for non‐contrast (NC) and contrast‐enhanced (CE) Head (a), Chest (b), and Abdomen‐pelvis (c) CT protocols. The solid blue lines indicate the mean bias, and the dashed red lines indicate the 95% limits of agreement (LoA). Color‐coded shaded regions represent the 95% confidence for the biases and LoA.

Bland‐Altman analysis of $D_{w}$ accuracy and precision demonstrated mean negative biases ranging from −0.1% to −2.0% across all protocols with LoA ranging from −6.4% to 3.2% (Figure 2, Table 1). This underestimation was more pronounced in the Head and Chest (−2.0% to −0.8%) body regions compared to Abdomen‐pelvis (−0.2% to −0.1%), suggesting that body region impacts the degree of systematic $D_{w}$ underestimation. However, the 95% LoA was entirely at or below zero for only the NC and CE Head protocols indicating that consistent underestimation of $D_{w}$ was only observed in the head. The widest LoA were observed for the CE Chest protocol (−6.4% to 2.9%) compared to the NC Head protocol which resulted in the narrowest LoA (−2.4% to 0.0%). $D_{w}$ bias differed significantly between the NC and CE protocols in the Head (*p*_Mann‐Whitney_ < 0.001) and Chest (*p*_Mann‐Whitney_ = 0.030) body regions. However, given the RMSE per protocol remained below the acceptable threshold of 10%, these statistically significant differences are not clinically significant.

Statistically significant proportional bias was identified for the CE Abdomen‐pelvis protocol (*p* = 0.018). However, the regression coefficient was small (0.10%/cm) and R^2^ = 0.185 indicating that patient size explained a negligible proportion of variance in the $D_{w}$ error.

### 3.3 SSDE

Accuracy (bias) and precision (LoA) in derived SSDE values are summarized in Table 4. The maximum ARE of SSDE values per series was below 10% for all protocols, ranging from 2.7% to 7.0%, well within the clinically acceptable 20% threshold provided in AAPM TG‐204 and TG‐293. Consistent with the results observed for $D_{w}$, systematic SSDE bias was statistically significant in the Head and Chest body regions (*p *< 0.001), but bias did not reach statistical significance for the Abdomen‐pelvis protocols. Additionally, effect size was large for the Head and Chest body regions (*r* range: 0.55 – 0.87) and negligible for the Abdomen‐pelvis (*r* < 0.02). These effect size results are again consistent with the pattern observed for $D_{w}.$

**TABLE 4:** Accuracy (bias) and precision (limits of agreement) of size‐specific dose estimates derived using 3D landmark‐based $D_{w}$ estimations relative to the reference diagnostic CT series, summarized by protocol.

Protocol	Mean Bias [95% CI] (%)	Median Bias (%)	*p*‐value^*^	Effect Size	Max ARE (%)	Lower LoA [95% CI] (%)	Upper LoA [95% CI] (%)
**NC Head**	1.1 [0.9, 1.2]	1.0	<0.001	0.87	2.7	0.0 [−0.3, 0.2]	2.2 [1.9, 2.5]
**CE Head**	1.9 [1.5, 2.2]	1.8	<0.001	0.87	3.1	0.0 [−0.6, 0.6]	3.7 [3.1, 4.3]
**NC Chest**	0.7 [0.3, 1.1]	0.4	<0.001	0.55	3.6	−1.6 [−2.3, −0.9]	3.0 [2.3, 3.7]
**CE Chest**	1.8 [0.9, 2.6]	1.9	<0.001	0.62	7.0	−2.8 [−4.3, −1.3]	6.3 [4.8, 7.8]
**NC Abd‐pel**	0.0 [−0.5, 0.6]	0.0	0.964	0.01	5.0	−3.6 [−4.5, −2.6]	3.6 [2.7, 4.6]
**CE Abd‐pel**	0.0 [−0.4, 0.5]	−0.2	1.00	0.00	3.4	−2.5 [−3.3, −1.7]	2.8 [1.7, 3.4]

Abbreviations: ARE, absolute relative error; CE, contrast‐enhanced; CI, confidence interval; LoA, limits of agreement; NC, non‐contrast.

* Test whether median bias SSDE bias differs significantly from zero using the Wilcoxon signed‐rank test.

**FIGURE 3:**
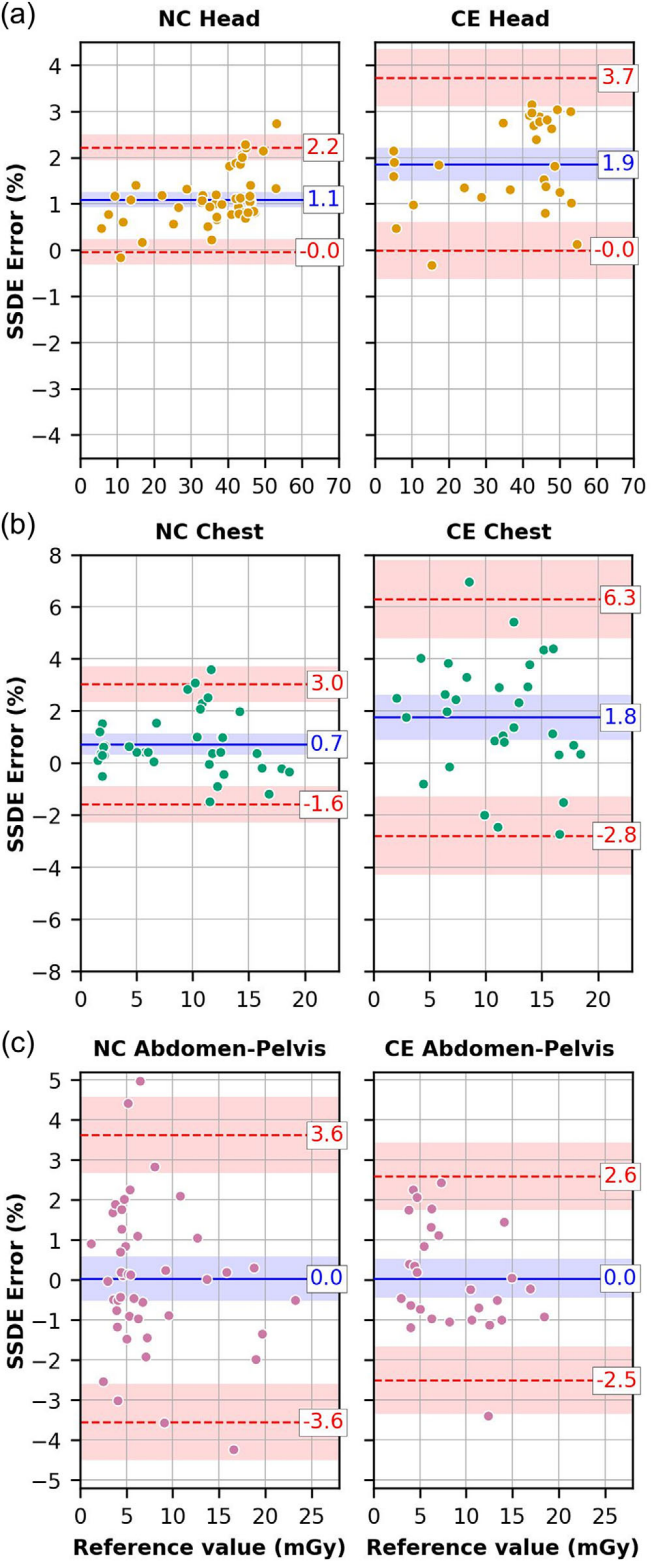
Bland‐Altman plots depicting the size‐specific dose estimate (SSDE) relative error, expressed as a percentage, for the 3D landmark (3DLM) method compared to reference diagnostic CT measurements for non‐contrast (NC) and contrast‐enhanced (CE) Head (a), Chest (b), and Abdomen‐pelvis (c) CT protocols. The solid blue lines indicate the mean bias, and the dashed red lines indicate the 95% limits of agreement (LoA). Color‐coded shaded regions represent the 95% confidence for the biases and LoA.

Bland‐Altman analysis revealed mean SSDE biases ranging from 0.0% to 1.9% across protocols, and LoA ranging from −3.6% to 6.3% (Figure 3, Table 4). Mean SSDE bias was consistently positive for the Head and Chest body regions (0.7% to 1.9%) and near zero for Abdomen‐pelvis (0.0%). These findings are expected given the observed underestimation of $D_{w}$ for the Head and Chest body regions (Figure 2, Table 3) which, by definition, increases the SSDE value. The CE Chest protocol also demonstrated the widest LoA (−2.8% to 6.3%), as shown in Figure 3b, which is expected given the pattern observed for $D_{w}$.

The bias of 3DLM‐derived SSDE values differed significantly between NC and CE series for the Head (*p*_Mann‐Whitney_ < 0.001) and Chest (*p*_Mann‐Whitney_ = 0.016) body regions, and no significant difference was observed for the Abdomen‐pelvis body region. Despite the observed differences between NC and CE series for both the Head and Chest body region, given the maximum ARE of SSDE values per series remained below the acceptable threshold of 20%, these differences are not clinically significant.

**FIGURE 4:**
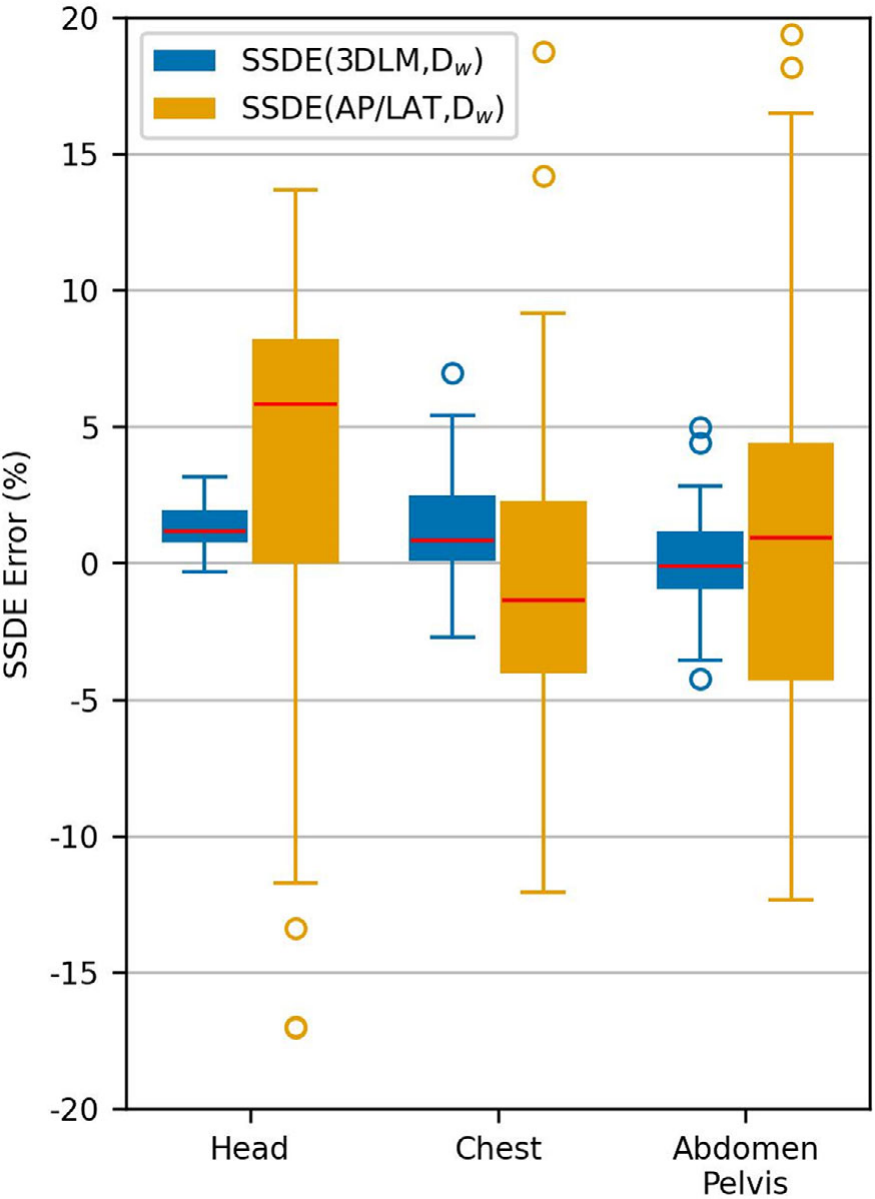
Box plots depicting the size‐specific dose estimate (SSDE) relative error, expressed as a percentage, using water equivalent diameter ($D_{w}$) measured directly in 3D landmark (3DLM) images “SSDE(3DLM, $D_{w}$)” and a conventional approach using $D_{w}$ derived from scanner‐reported anterior‐posterior (AP) and lateral (LAT) dimensions “SSDE(AP/LAT, $D_{w}$)”. Each method is represented separately by color‐coding and grouped by body region, with contrast‐enhanced and non‐contrast diagnostic CT protocols pooled for each comparison. Each box represents the interquartile range (IQR), the central red line indicates the median error, the whiskers extend to 1.5× IQR, and individual data points beyond the whiskers represent outliers.

Figure 4 depicts grouped box plots of the SSDE error for the new method “SSDE(3DLM, $D_{w}$)” and conventional method “SSDE(AP/LAT, $\;D_{w}$)” relative to the reference method using the diagnostic CT series. The new method provided significantly lower median SSDE bias (*p* < 0.001) compared to the conventional method for the Head (1.2% and 5.8%, respectively) and Chest (0.8% and −1.4%, respectively) body regions with NC and CE series pooled within each region. No significant difference was observed for the Abdomen‐pelvis (*p* = 0.350; −0.1% and 0.9%, respectively). As illustrated in Figure 4, variability in the per‐series SSDE error was significantly lower for the new method across all body regions (*p_Levene_ *< 0.001) with the standard deviation (SD) ranging from 0.8% to 1.9% for SSDE(3DLM, $D_{w}$) and from 5.6% to 6.9% for SSDE(AP/LAT, $D_{w}$). The maximum ARE of SSDE values per series was also consistently lower for SSDE(3DLM, $D_{w}$) compared to SSDE(AP/LAT, $D_{w}$) with values of 3.1% and 17.0% for Head, respectively, 7.0% and 18.7% for Chest, and 5.0% and 19.4% for Abdomen‐pelvis.

## 4 DISCUSSION

3D landmark series were found to provide clinically acceptable estimations of $D_{w}$ when compared to reference diagnostic non‐contrast and contrast‐enhanced CT series across a wide range of patient ages, patient sizes, and body regions. The RMSE of $D_{w}$ measurements per protocol was less than 3% across all comparisons which is well within the clinically acceptable threshold of 10% recommended by AAPM TG 220 report where the concept of $D_{w}$ was originally introduced. SSDE values derived from 3DLM‐based $\;D_{w}$ measurements were also found to provide per series ARE values less than 7.1% which is considerably lower than the clinically acceptable threshold of 20% reported by AAPM TG 204 and 293 where reference body and head CTDI_vol_‐to‐SSDE conversion factors were originally established.

Beyond confirming that the present results remained below the acceptable error measures from AAPM TG reports, generalizability to broader patient populations was assessed by comparing $D_{w}$ ranges with those reported in prior studies. McCollough *et al.* ^14^ utilized a cohort of 250 NC adult and pediatric CT scans, and the $D_{w}$ ranges were similar across all three body regions: 12.6 – 20.9 cm (present study) vs. 14.6 – 17.8 cm for Head, 9.8 – 34.6 cm vs. 12.0 – 36.0 cm for Chest, and 12.7 – 41.6 cm vs. 9.5 – 42 cm for Abdomen‐pelvis. Results of the present study were also compared against established diagnostic reference levels^15^ (DRLs) for adult Chest and Abdomen‐pelvis CT scans as these are the only regions for which Kanal et al. reported $D_{w}$‐based DRLs. The $D_{w}$ values in the present study were well represented by the range of values reported from the ACR Dose Index Registry (21 – 41 cm for Adult Chest and Abdomen‐pelvis). This agreement with both a matched single‐site cohort and a national registry indicates that the patient size distribution captured in this study is representative of the broader clinical population. Together these results indicate that 3DLM scans can be utilized beyond their intended purpose to support prospective and accurate estimations of $D_{w}$ and SSDE.

Bland‐Altman analysis revealed a negative $D_{w}$ bias across all protocols, but consistent underestimation of $D_{w}$ was observed only for the Head body region, as evidenced by 95% LoA entirely at or below zero for both NC and CE Head protocols (Figure 2). This consistent underestimation may reflect inherent beam quality differences between the 3DLM Ag filtration and the Cu filtration used for the diagnostic CT acquisitions. Spectral modeling confirmed a higher effective energy for the 120 kV/Ag spectrum (82 keV) relative to the 120 kV/Cu spectrum (57 keV). The higher effective energy of the 3DLM Ag filtration reduces the photoelectric contribution to total attenuation in high Z materials such as bone. This reduction disproportionately lowers the CT number for bone relative to soft tissue and reduces the estimated $D_{w}$ in Head CT protocols, where bone comprises a significant portion of the imaged anatomy. This effect likely contributed to the consistent underestimation of $D_{w}$ observed in the Head body region. In addition, the underestimation was significantly higher and the LoA were larger for the CE Head protocol compared to the NC Head protocols, which may be partially attributable to the influence of iodinated contrast on the CT number of soft tissue. The larger LoA for the CE Head protocol are likely driven by timing variability. Delayed phase acquisitions (CT head with contrast) constituted 43% of the CE series, while arterial phase acquisitions (CTA Head) made up the remaining 57%, resulting in differing iodine enhancement and, consequently, greater variability in the amount of contrast present. The combination of the influence of contrast, contrast timing differences, and filtration‐related beam quality differences may further amplify the $D_{w}$ underestimation and overall higher variability for the CE Head protocol.

Comparable trends were observed for the Chest body region, the $D_{w}$ bias became more negative and the LoA increased for the CE Chest protocol compared to NC. This finding also likely reflects contrast timing variability as arterial phase acquisitions (CTA Chest) constituted 83% of the CE Chest series, while later arterial phase acquisitions made up the remaining 17%, resulting again in differing iodine enhancement and greater variability in the amount of contrast present.

Abdomen‐pelvis trends were somewhat unique. $D_{w}$ bias did not differ significantly between the CE and NC protocols, although the LoA decreased moderately for CE compared to NC. This behavior is likely because most CE Abdomen‐pelvis series (63%) were acquired in the portal venous phase rather than the arterial phase, which involves relatively less iodine enhancement.

The positive SSDE bias reported for the Head and Chest body regions is concordant with the propagation of $D_{w}$ underestimation through the size‐based CTDI_vol_‐to‐SSDE conversion factors. However, consistent underestimation of SSDE was observed only for the Head body region, as evidenced by 95% LoA entirely at or above zero for both NC and CE Head protocols (Figure 3). A significantly higher SSDE overestimation was also observed for the CE Head protocols compared to the NC Head protocols. These differences are likely attributed to the same effects described above for $D_{w}$.

There are other potential reasons for the small, but measurable biases observed for the Head and Chest regions. Cases were not excluded or screened for the presence of metal implants, so implants could have been present in some scans. Hip and pelvic hardware are some of the most common implants encountered on CT images. However, $D_{w}$ bias for the Abdomen‐pelvis protocol remained low, with the 95% CI falling entirely within ±1%, indicating any such impact was not substantial. The manufacturer also did not disclose if a beam hardening correction is applied to the 3DLM scans. A 120 kV/Ag spectrum has a considerably higher effective energy meaning it already contains fewer low‐energy photons that would otherwise be preferentially absorbed by tissue, which may reduce the potential for beam hardening artifact even without correction. If a correction is absent, residual cupping artifacts could still compound the beam‐quality effects discussed above, particularly in the head. In addition, table attenuation can contribute proportionally more when imaging smaller patients. While our analysis did not directly test for this effect, the proportional bias analysis partly addresses it. Bias was significant only for the Abdomen‐pelvis protocol, but the small regression coefficient and R^2^ indicate no meaningful dependence of $D_{w}$ error on patient size, including in pediatric patients. This finding further suggests the 3DLM method performs consistently across the range of patient sizes encountered in clinical practice. Although statistically significant $D_{w}$ and SSDE biases were observed for the Head and Chest body regions, the differences are not clinically significant, as RMSE values of $D_{w}$ measurements per protocol were < 3% and ARE values of SSDE(3DLM, $D_{w}$) per series were all < 7.1%, well within the acceptable thresholds of 10% and 20%, respectively.

The new method for SSDE estimation, SSDE(3DLM, $D_{w}$), demonstrated superior performance across all body regions when compared to the conventional method, SSDE(AP/LAT, $D_{w}$). It is unclear whether the scanner‐reported AP/LAT dimensions used for comparison in this study are derived from the 3DLM volume or synthesized AP/LAT projections given this is proprietary information. Any resulting inaccuracy could propagate to the SSDE values compared here. As a secondary, exploratory check, the known AP/LAT dimensions of three uniform image quality phantoms (20 cm diameter cylinder; 24 cm x 28 and 28 cm x 32 cm elliptical) were compared to scanner‐reported values. Mean absolute differences of 4.4% and 5.6% were found, respectively. A full validation of scanner‐reported AP/LAT accuracy is beyond the scope of this study. The statistically significant reduction in systematic SSDE bias observed in the Head and Chest body regions (Figure 4) reflects the associated improvement in $D_{w}$ accuracy using the 3DLM method given that the error in the $D_{w}$ estimation propagates directly into SSDE through the size‐based conversion factors. In addition, the lower per‐series variability in SSDE error across all body regions indicates that the 3DLM method provides more reproducible estimates.

The present study has several limitations. First, the results were based on measurements from a single institution using a single CT system which may limit the generalizability of these findings across different sites and scanners. However, the underlying method for $D_{w}$ estimations which relates CT number to patient attenuation properties is a generalizable approach applicable across CT platforms. It is the specific beam quality of the 120 kV /Ag spectrum used in the 3DLM acquisitions, and characterized here, that is unique to this manufacturer's configuration and would need to be reassessed on other systems. Second, the conversion from $D_{eff}$ to $D_{w}$ applied in this study for comparing SSDE using the new 3DLM‐based method relative to the conventional method was derived from published pediatric patient data.^10^ However the size range of the conversion data is consistent with the size range of the adult patient cohort included in this study. Third, $D_{w}$ was measured in the central axial slice of the diagnostic CT series rather than averaged across the entire scan length which may not fully capture size variation along the craniocaudal axis. The primary aim of this study was to validate the accuracy of 3DLM‐based $D_{w}$ estimation as the foundation for future clinical implementation. Extension to the full scan length could be readily incorporated by the manufacturer without any anticipated degradation in performance. Fourth, in isolated cases where patient motion between acquisitions resulted in clear anatomical misalignment, the reference central axial slice for the 3DLM‐based $D_{w}$ estimation was adjusted to maintain consistent anatomical position for comparison. The frequency of these adjustments was small and their impact on the overall results is expected to be negligible.

## 5 CONCLUSION

The findings from this study confirm that 3DLM‐based estimates of $D_{w}$ and SSDE achieve clinically robust accuracy and precision across all six CT protocols evaluated, with $D_{w}$ RMSE values well below the 10% TG‐220 threshold and SSDE ARE values well below the 20% threshold recommended by TG‐204 and TG‐293. Systematic bias was consistently small across all protocols and showed negligible dependence on patient size confirming consistent performance across the range of patient sizes encountered in this study. The new 3DLM‐based method also demonstrated superior SSDE estimation accuracy and reproducibility when compared to a conventional method using $D_{w}$‐derived from the AP/LAT dimensions reported by the scanner.

This work supports 3DLM‐based $D_{w}$ estimation as a practical and reliable approach for SSDE derivation, extending the utility of 3DLM acquisitions beyond their intended purpose to provide prospective, accurate SSDE determination. We anticipate that adapting manufacturer software to support this capability is relatively straightforward. The method also serves as a practical alternative to diagnostic CT‐derived measurements with direct applicability to both retrospective dose reporting as well as prospective dose estimations. Accurate and reproducible prospective $D_{w}$ estimation from 3DLM acquisitions may also inform future development in automated, size‐based TCM selection, potentially reducing reliance on manual size‐specific protocoling.

## AUTHOR CONTRIBUTIONS

Andrew M. Hernandez conceived of the study, designed the experiments, acquired the data, analyzed the results, and wrote the manuscript. Sarah Yoon processed the data, analyzed the data, and edited the manuscript. Michael T. Corwin helped design the methodology, edited the manuscript, and provided clinical expertise. Eric S. Diaz helped design the methodology, edited the manuscript, and provided clinical expertise. Ahmadreza Ghasemiesfe provided clinical expertise and edited the manuscript. Sarah E. McKenney helped design the methodology, drafted and edit the manuscript. John M. Boone helped design the research question, edited and reviewed the manuscript for clarity.

## CONFLICT OF INTEREST STATEMENT

The CT scanner used in this study was provided, in part, through a research cooperation agreement between Canon Medical Systems USA and the UC Davis Health Department of Radiology. This agreement is unrelated to the present study, and Canon personnel had no role in study design, data collection, analysis, or interpretation of findings. Ahmadreza Ghasemiesfe and Eric S. Diaz have received speaker honoraria from Canon Medical Systems USA. Sarah E. McKenney manages the institutional research agreement with Canon Medical Systems USA and receives monetary and in‐kind support through it, unrelated to this work.

## ETHICS STATEMENT

This retrospective study of standard‐of‐care images received an exemption determination from the Institutional Review Board.

## Supporting information




Supporting Information

## Data Availability

The data supporting the findings presented in this study are not publicly available due to patient privacy and institutional data‐sharing policies.
