# Supplementary material for: Prospective estimation of water equivalent diameter and SSDE using 3D landmark CT scans
Source: J Appl Clin Med Phys. 2026 Aug 24;27(9):e70742. doi: 10.1002/acm2.70742 (PMC13504162; doi:10.1002/acm2.70742)
Supplement: Supplementary file 1 [file ACM2-27-e70742-s001.docx]

**Supplemental Methods**

**X-ray Beam Spectral Modeling and Quality Characterization**

To evaluate the degree of beam quality differences between the 120 kV / Ag-filtered beam for the 3DLM ascquisition and the 120 kV / Cu-filtered beam used for the diagnostic CT acquisitions, spectra were modeled using TASMICS (Tungsten Anode Spectral Model using Interpolating Cubic Splines) [15]. Previously reported filter thicknesses for the Ag (0.5 mm) and Cu filtration (0.36 mm) at 120 kV were used in the modeling [2], and no other additional filtration was included.

The half-value layer and effective energy were derived from each modeled spectrum using standard attenuation-based methods. Resulting values were 8.57 mm Al and 57 keV, respectively, for the 120 kV/Cu spectrum, and 12.67 mm Al and 80 keV, respectively, for the 120 kV/Ag spectrum.

**Figure S1.** TASMICS-modeled x-ray spectrum for the 120 kV / Cu and 120 kV / Ag beams for the Canon Aquilion One Insight Edition CT scanner.
